# Greening Reversed-Phase Liquid Chromatography Methods Using Alternative Solvents for Pharmaceutical Analysis

**DOI:** 10.3390/molecules23051065

**Published:** 2018-05-02

**Authors:** Moussa Yabré, Ludivine Ferey, Issa Touridomon Somé, Karen Gaudin

**Affiliations:** 1 ChemBioPharm Team, ARNA Laboratory, INSERM U1212, CNRS UMR 5320, Bordeaux University, F-33000 Bordeaux, France; moussa.yabre@u-bordeaux.fr (M.Y.); karen.gaudin@u-bordeaux.fr (K.G.); 2 Laboratoire de développement du médicament, Université Ouaga 1 Pr Joseph Ki-Zerbo, Ouaga 03 BP 7021, Burkina Faso; tsome@ulb.ac.be

**Keywords:** green liquid chromatography, reversed-phase chromatography, alternative solvents, pharmaceutical analysis, ethanol, micellar liquid chromatography, ionic liquids

## Abstract

The greening of analytical methods has gained increasing interest in the field of pharmaceutical analysis to reduce environmental impacts and improve the health safety of analysts. Reversed-phase high-performance liquid chromatography (RP-HPLC) is the most widely used analytical technique involved in pharmaceutical drug development and manufacturing, such as the quality control of bulk drugs and pharmaceutical formulations, as well as the analysis of drugs in biological samples. However, RP-HPLC methods commonly use large amounts of organic solvents and generate high quantities of waste to be disposed, leading to some issues in terms of ecological impact and operator safety. In this context, greening HPLC methods is becoming highly desirable. One strategy to reduce the impact of hazardous solvents is to replace classically used organic solvents (i.e., acetonitrile and methanol) with greener ones. So far, ethanol has been the most often used alternative organic solvent. Others strategies have followed, such as the use of totally aqueous mobile phases, micellar liquid chromatography, and ionic liquids. These approaches have been well developed, as they do not require equipment investments and are rather economical. This review describes and critically discusses the recent advances in greening RP-HPLC methods dedicated to pharmaceutical analysis based on the use of alternative solvents.

## 1. Introduction

High-performance liquid chromatography (HPLC) is the most widely used analytical tool for pharmaceutical analysis. Indeed, it is the most important technique involved in the quality control of bulk drugs and pharmaceutical formulations (i.e., analysis of active pharmaceutical ingredients (API), characterization of impurities, determination of degradation products to test product stability, and determination of enantiomeric purity), and also in the determination of drugs and metabolites in biological samples [1]. Most HPLC methods developed in pharmaceutical laboratories are based on the reversed-phase (RP) mode, using a hydrophobic stationary phase and a polar mobile phase. In quality control, the primarily used detection mode is ultraviolet (UV)/Visible detector. Therefore, mobile phase compatibility with this detection is a parameter often taken into account in pharmaceutical analysis development. The mobile phase of RP-HPLC is usually a mixture of water (containing additives to adjust pH and ionic strength) and organic solvent, such as acetonitrile (ACN) and methanol (MeOH) [2]. These two solvents are by far the preferred organic solvents used in RP-HPLC because of their remarkable combination of properties favorable for RP-HPLC applications. Among them include complete miscibility with water, relatively low viscosity of their aqueous solutions (especially in the case of ACN), low UV cut-off wavelength (190 nm and 205 nm for ACN and MeOH, respectively), availability in the high purity required for HPLC, and low chemical reactivity with most sample species, as well as with HPLC instrument and column surfaces [2,3].

Despite these remarkable properties, ACN and MeOH present some issues in terms of environmental impact and health safety. Indeed, ACN is flammable, volatile, and toxic. Even if MeOH is less toxic and more easily biodegradable than ACN, it is also ranked as a hazardous solvent due to its inherent toxicity and the great requirements of its waste disposal [4,5]. Unfortunately, the amount of waste generated by RP-HPLC analyses cannot be neglected. In fact, one continuously operating liquid chromatograph equipped with a conventional LC column (15–25 cm length, 4.6 mm i.d., packed with 5 µm particles) and a mobile phase flow rate of 1 mL/min produces about 1.5 L of waste per day, meaning about 500 L of effluent per year [3]. Although this volume of waste is small compared to the amount of waste generated by large industrial manufacturing companies, some big pharmaceutical companies use hundreds of liquid chromatographs in their research and development, and quality control laboratories, resulting in thousands of liters of toxic waste produced every day. Moreover, the use of HPLC is becoming more and more intensive due to the technological advances allowing high-throughput analysis, which also increases, at the same time, the amount of waste produced. These HPLC waste streams containing ACN and MeOH must be disposed as chemical waste, which is costly and adds to the environmental waste-disposal burden of the laboratory.

Regarding the health and environmental issues of organic solvents commonly used in RP-HPLC, the greening of RP-HPLC methods has received great interest in the analytical community, whose aim is to search for new alternatives to replace polluting analytical methods with cleaner ones. Green analytical chemistry (GAC) emerged from green chemistry in the 2000s [6,7], and has gained increasing attention and acceptance among researchers. Its concept refers to eliminating or reducing hazardous chemicals from analytical processes to improve environmental and health friendliness, without compromising method performance [8].

Based on the 12 principles of green chemistry, some strategies are commonly implemented to achieve greener liquid chromatography methods. They focus on a reduction in solvent consumption through a decrease in column length, internal diameter, and/or column particle; the replacement of toxic and hazardous solvents, such as ACN and MeOH, with less toxic and more environmentally friendly alternatives; and by increasing the importance of recycling in larger scale preparative separation technologies [3,9]. Chromatographic techniques have the potential to be greener at all steps of the analysis, from sample collection and preparation, to separation and final determination. Some strategies for greening chromatographic methods are more efficient than others; therefore, evaluation methods are needed to assess the greenness of analytical methods. Some tools have already been developed; the two best known are the NEMI labelling and analytical Eco-scale methods [10,11,12,13]. NEMI labelling results in an easy-to-read pictogram stating if hazardous or corrosive reagents are used or if the procedure generates significant amounts of waste [10]. The analytical Eco-scale is a more quantitative approach, based on subtracting penalty points from a total of 100, based on the amount and hazard of reagents, energy consumption, occupational hazards, and amount of waste generated [11].

Over the past years, several reviews have covered the application of GAC principles to chromatography analysis in general [3,9,14,15], while some focused especially on pharmaceutical analysis [16,17,18,19]. Since it is difficult to eliminate the use of organic solvents in RP-HPLC, the better way to make this technique greener is to replace hazardous solvents with more benign ones. Solvent-reduction strategies can also be successfully followed to achieve a method even more ecological. However, an overall reduction in the amount of solvent used, and consequently the waste generated, often implies the purchase of expensive ultra-high-performance liquid chromatography (UHPLC) instruments or the development of new technologies. This review will present recent advances in greening RP-HPLC methods dedicated to pharmaceutical analysis through the use of alternative solvents.

## 2. Alternative Organic Solvents in RP-HPLC

Mobile phases in RP-HPLC are classically mixtures of water, containing additives to adjust pH and ionic strength, and organic solvent. Acetonitrile and MeOH are the two organic modifiers most widely used by HPLC users in the RP. Unfortunately, both solvents are ranked hazardous due to their toxic effect and the great importance placed on the safe detoxification of their waste, even though MeOH is considered more environmentally friendly than ACN, and therefore, should be preferred whenever possible [5]. Since it appears difficult to develop a RP-HPLC method without an organic solvent, a strategy to make this technique greener is to replace ACN and MeOH with other less toxic organic solvents to minimize the environmental and health impacts. The greenness degree of an organic solvent is assessed based on its environmental, health, and safety (EHS) criterion and life-cycle assessment (LCA) [20]. The EHS assessment is composed of environmental indicators (e.g., water and air hazards, and persistency), as well as indicators related to human health (e.g., chronic and acute toxicity, and irritation) and safety (e.g., stability, reactivity, flammability, explosion, and release potential) hazards. It allows the assessment of possible hazards inherent to the solvent properties. The LCA method is used for a detailed assessment of emissions into the environment, as well as resource use over the full life cycle of a solvent. It includes the production, use, potential recycling, and disposal of a solvent. In other words, LCA allows the quantification of environmental impacts that are indirectly attributable to the use of solvents. An organic solvent will be preferable in terms of greenness if it combines good EHS and LCA assessments. Several general solvent selection guides (SSGs), based on EHS only or EHS combined with LCA, have been published [20,21,22,23,24]. The SSGs have mainly been developed by pharmaceutical industry companies, and they present some differences in the classification of solvents which can be considered as green [23]. These guides have been largely developed to be applied to organic synthesis, and thus, are not adapted to solvent selection for analytical chemistry applications [25]. The organic solvents commonly accepted as green, and which can be used in RP-HPLC, are ethanol, isopropanol, n-propanol, acetone, ethyl acetate, ethyl lactate, and propylene carbonate [25,26,27].

### 2.1. Ethanol

#### 2.1.1. Chromatographic Properties

Ethanol (EtOH) is one of the greenest organic solvents, which makes it a particularly desirable solvent for green liquid chromatography [9]. Compared to ACN and MeOH, EtOH is less toxic and has a lower vapor pressure, which leads to less evaporation, and consequently, to lower inhaled quantities. EtOH is also widely available and less expensive (particularly EtOH 96%) than ACN and MeOH, which promotes its use in resource-limited laboratories, particularly in developing countries [3]. Moreover, due to its environmentally compatible waste, EtOH has lower disposal costs compared to ACN and MeOH, which is advantageous for sensitive areas where chemical-waste disposal is still too expensive.

From a chromatographic point of view, EtOH has similar properties to ACN and MeOH. For example, Miyabe et al. [28] studied the adsorption characteristics in RP-HPLC of an EtOH/water mixture on an octadecylsilyl (ODS)-silica gel column, and compared them to corresponding results obtained from MeOH/water and ACN/water mixture-based mobile phases. In the three chromatographic systems, they found a similar mechanism of surface diffusion with regard to enthalpy-entropy compensation and linear, free-energy relation. Based on this study, quite similar contributions of adsorption mechanisms were demonstrated. From this point of view, similar separation mechanisms should be expected using these different solvent mixtures. Also, EtOH/water mixtures were evaluated by Ribeiro et al. [29], as reversed-phase mobile phases on both C8 and C18 columns. They showed that, by substituting ACN or MeOH with EtOH as a green modifier, similar peak efficiency was obtained for the chromatographic separation of a mixture containing neutral and basic compounds. As confirmed later by other investigators, satisfactory performance could be achieved with EtOH [30]. In terms of selectivity, EtOH is in the same group as MeOH, according to Snyder et al.’s [2] classification of organic solvents, which means that they have a similar proton acceptor, proton donor, and dipole moments. In addition, EtOH has a higher eluotropic strength, which means that a lower percentage of EtOH than MeOH is needed in the mobile phase for comparable retention times [26].

However, EtOH has two main drawbacks which can hinder its use in RP-HPLC [16]. The first drawback is that the UV cut-off of EtOH (210 nm) is higher than that of MeOH and ACN [2], which can result in elevated background noise and an important drift of baseline when gradient elution is used, leading to a reduction in sensitivity when UV detection is hyphenated. Nevertheless, the high UV cut-off of EtOH may not be a problem if compounds have strong UV chromophores, or if mass-spectrometry (MS) detection is used. The important number of applications reported in Table 1 proves that this limitation can be overcome. The second drawback comes from the viscosity of EtOH/water mixtures, which is higher than that of MeOH/water and ACN/water mixtures for equivalent eluotropic strength at room temperature. Such high viscosity leads to high backpressures, and thus, limits the use of EtOH with conventional LC systems (400 bar) [17]. The main strategy to overcome the high pressures generated by viscous mobile phases is the use of UHPLC systems, which can support high pressures above 1000 bar. However, even if UHPLC instruments are widely available on the market, these instruments are expensive, and thus, less accessible to limited-resource laboratories.

Another way to overcome the high pressure generated by ethanol-based mobile phases is the use of columns packed with superficially porous particles, which are also compatible with conventional LC instruments. The development of columns packed with such particles was considered a breakthrough in column technology, since it limits backpressures created, and thus, allows the use of high mobile phase flow rates, while maintaining column efficiencies [31]. The low pressures generated by these columns also offer the possibility of the use of mobile phases with higher-viscosity EtOH/water mixtures.

Performing LC separations at elevated mobile phase temperatures, known as high-temperature liquid chromatography (HTLC), is also a way to reduce viscosity of ethanol-based mobile phases. It is well known that the temperature of the column affects selectivity, efficiency, and mobile phase viscosity. By increasing the temperature, the viscosity of the mobile phase decreases, resulting in reduced backpressures. For example, solvent viscosity can decrease by about 50% by raising the temperature from ambient (20 °C) to about 50 °C [32]. Many interesting reviews about HTLC have been published [33,34,35,36,37]. To successfully implement high temperatures in LC, special instrumentation is required, such as a mobile phase preheater, a column heater, and a post-column, effluent cooling system [34]. Although HTLC brings many advantages, it also has limitations. One of them is the thermal stability of stationary phases. Indeed, for most silica-based columns used in reversed-phase conditions, the maximum allowed temperature should not exceed 60 °C [35]. Another limitation is that analyzed compounds must be thermally stable. At the end, the use of high column temperatures induces an increase in energy consumption, which is in contradiction with GAC principles [7].

#### 2.1.2. Pharmaceutical Applications

**Figure 1:**
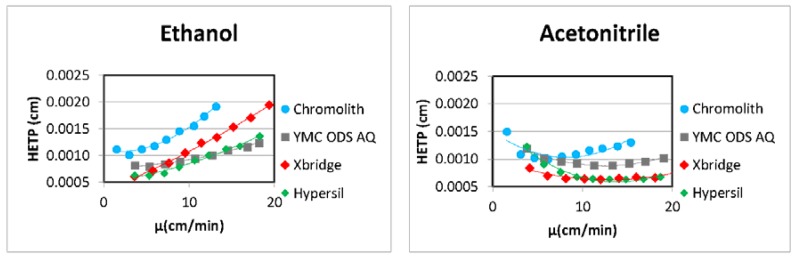
Comparison of Van Deemter curves of atorvastatin obtained using ethanol or acetonitrile in mobile phases. Xbridge BEH Shield RP18 (50 × 4.6 mm i.d., 2.5 µm); Chromolith (SpeedROD RP18, 50 × 4.6 mm i.d., 2 µm); YMC-ODS-AQ (50 × 4.6 mm i.d., 3 µm); Hypersil GOLD (50 × 4.6 mm i.d., 1.9 µm). Isoeluotropic mobile phases: EtOH/formic acid (pH 2.5, 25 mM) in proportions 40/60 (*v*/*v*) for YMC column; 38/62 (*v*/*v*) for Xbridge and Hypersil columns; and 35/65 (*v*/*v*) for Chromolith column. HETP: height equivalent to a theoretical plate. Similar to Reference [42].

Green RP-HPLC methods using ethanol-based mobile phases have been widely reported for the analysis of drugs in pharmaceutical formulations [38,39,40,41,42,43,44,45,46,47,48,49,50,51,52,53,54], as well as in biological samples [55,56] (Table 1). For example, a HPLC/diode-array detector (DAD) method using EtOH as the organic solvent in the mobile phase and an octadecyl-grafted silica column, was developed and validated by our team for the analysis of statins (i.e., pravastatin, fluvastatin, and atorvastatin in high concentrated EtOH/water solutions) [42]. The mobile phase was composed of an EtOH/aqueous solution of formic acid (pH 2.5, 25 mM) (50:50, *v*/*v*) at 40 °C. During method development, four columns with particle sizes between 1.9 µm and 3 µm were compared regarding efficiency using Van Deemter plots. For these acidic compounds, the benefit of keeping efficiency within a large range of flow rate, classically achieved through columns with particle sizes under 2 µm, was not observed with ethanol-based mobile phases compared to acetonitrile ones (Figure 1). Such loss of efficiency with the flow rate may be a limit of the use of EtOH in some cases.

**Figure 2:**
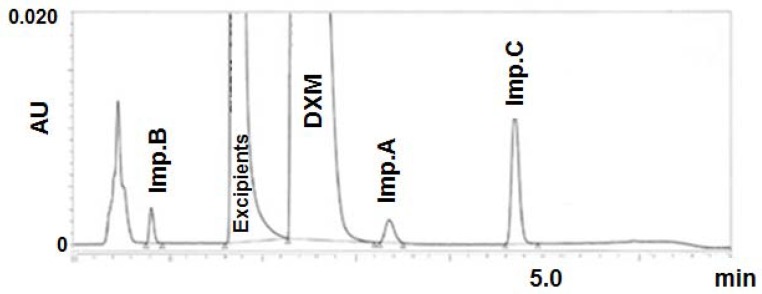
Reversed-phase high-performance liquid chromatography (RP-HPLC) chromatogram of a mixture of dextromethorphan (DXM), its impurities, and excipients. Acquity BEH C18 column (50 × 2.1 mm i.d, 1.7 µm). Gradient elution of ethanol (slope of 3.1%/min) with 10 mM ammonium formate adjusted at pH 4.7 with formic acid. Flow rate: 0.23 mL/min. Temperature: 38.0 °C. Detection at 280 nm. Similar to Reference [44].

Elzanfaly et al. [45] developed and validated green HPLC methods, with mobile phases composed of EtOH/water mixtures at room temperature, for the analysis of coformulated pharmaceuticals treating gastrointestinal tract disorders: clidinium bromide/chlordiazepoxide hydrochloride, phenobarbitone/pipenzolate bromide, mebeverine hydrochloride/sulpiride, and chlorphenoxamine hydrochloride/caffeine/8-chlorotheophylline, either in their bulk powder or in their dosage forms. The developed methods were compared to the reported conventional HPLC methods and were found to be greener using the NEMI assessment [12]. More time— and solvent— saving was reported, hence these new methods can be used for routine analysis of the studied mixtures with a low impact on the environment. In another study, Rojanarata [38] developed a stability-indicating RP-HPLC method for the assay of prednisolone tablets using a mixture of EtOH/water (30:70, *v*/*v*) as the mobile phase. The separation was performed at 50 °C to overcome the increase in backpressure caused by the high viscosity of EtOH. Also, a green chromatographic method was developed and validated for the simultaneous determination of phenylephrine, paracetamol, and guaifenesin in their ternary pharmaceutical mixture using a mobile phase composed of EtOH and a phosphate buffer at pH 7.0 [47]. The separation was carried out on a monolithic column which allowed an efficient separation at higher flow rates, and consequently, a reduction in the analysis time. Lower quantities of solvent were consumed, and thus, a reduced amount of waste was produced. In another study, our team reported a green RP-HPLC method using a mobile phase composed of EtOH for stability studies of dextromethorphan [44]. The development was based on an innovative combination of green chemistry and Quality by Design (QbD) concepts to achieve a good quality of the method, while minimizing environmental impacts and analyst exposure. It is an example of how chemometrics can be used to aid in the development of green analytical chemistry. Experimental designs were used to identify parameters most affecting method performances, and to finally find the optimal operating conditions allowing the best chromatographic separation in terms of resolution between dextromethorphan and its degradation products, peak efficiencies, and solvent consumption. A response surface methodology allowed determining the Design Space (DS) (method operable design region), which was validated experimentally to prove method robustness inside. This method was validated using the concept of total error, and was used to analyze the main degradation products of dextromethorphan in a pharmaceutical product (Figure 2). The greenness of this method was also checked using the analytical Eco-scale tool.

**Figure 3:**
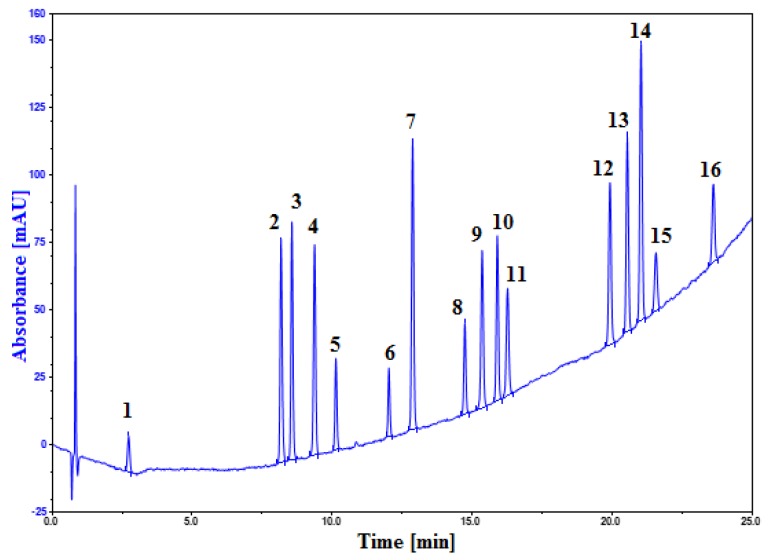
Separation of 16 active pharmaceutical ingredients. Xbridge BEH Shield RP18 column (50 × 4.6 mm i.d., 2.5 µm). Gradient elution of ethanol from 5% to 80% (slope of 2.57%/min) with acetate buffer 20 mM at pH 4.85. Flow rate: 0.8 mL/min. Temperature: 33.7 °C. Detection at 210 nm. Peak identification: 1 = paracetamol; 2 = doxylamine; 3 = ondansetron; 4 = melatonin; 5 = quinine; 6 = metopimazine; 7 = domperidone; 8 = pravastatin; 9 = ketoprofen; 10 = rosuvastatin; 11 = midazolam; 12 = diclofenac; 13 = atorvastatin; 14 = fluvastatin; 15 = artemether; and 16 = simvastatin. Similar to Reference [54].

Another application still in the field of pharmaceutical quality control, but this time dealing with multiproduct analysis, was recently published [54]. In this study, a specific and robust RP-HPLC method was successfully developed through QbD approach for the simultaneous separation of 16 active pharmaceutical ingredients (among them artemether without chromophore) using a gradient elution with EtOH as an organic modifier. Throughout the study, acetonitrile and ethanol-based mobile phases were investigated and compared. Optimal conditions could be found only with an ethanol-based mobile phase showing different selectivity between the two organic solvents (Figure 3). As shown in Figure 3, at 210 nm, a relatively high baseline drift was noticed in gradient mode using EtOH due to its high UV cut-off, however, artemether—a compound without chromophore in its structure—can still be detected.

In the field of bioanalysis, there are also some articles dealing with the use of EtOH as a green organic modifier in the mobile phase for the chromatographic analysis of pharmaceutical compounds [55,56]. For example, a HPLC assay was developed and validated by Hassanlou et al. [56] for the determination of capecitabine in human plasma using a C18 reversed-phase analytical column and a mobile phase composed of formic acid solution (pH 3):EtOH (55:45, *v*/*v*).

All these studies, using EtOH as organic modifier in RP-HPLC for pharmaceutical analysis, were carried out in the last decade, and deal with quality control and bioanalysis. This highlights anincreasing use of EtOH for chromatographic analysis at different stages of the pharmaceutical product lifecycle.

### 2.2. Propylene Carbonate

Propylene carbonate (PC)—a carbonate ester derived from propylene glycol—was reported as a possible ACN substitution in RP-HPLC [57,58]. It is a polar aprotic solvent mainly used as a reactive intermediate or as an inert solvent in various industries. It is used in degreasing, paint stripping, and cleaning applications, and as a carrier solvent for topically applied medications and cosmetics.

Propylene carbonate presents several advantages over can, such as a higher biodegradability, a lower toxicity, and a lower capacity to bio-accumulate, leading to a relatively easy disposal of PC waste [57]. Also, PC has a higher boiling point and flashpoint temperature, reducing the risk of accidental fires in laboratories. In addition, PC is commercially available as an HPLC-grade solvent at reasonable prices. However, PC has two major drawbacks which obviously limit the possibility of a direct transposition from ACN to PC in RP-HPLC [59,60]. The first drawback refers to the low miscibility of PC with water, which is why a third solvent is often added, such as MeOH or EtOH. The latter is the best candidate due to its green characteristics. The second problem comes from the high density and viscosity of PC, causing high-pressure drops in the chromatographic system. The backpressure will increase even more if EtOH is used as the ternary solvent.

Some applications of PC in RP-HPLC have been reported for the determination of active substances in pharmaceutical formulations, but most of them used MeOH as a ternary solvent [57,58,61,62,63]. Nevertheless, Tache et al. [59] studied the possibility of substituting ACN with PC and EtOH mixtures in RP-HPLC. Three series of compounds have been analyzed, including acidic (fenofibric, lovastatic, and simvastatic acids), neutral (toluene, fluorene, and fluoranthene), and basic (carbamazepine, diltiazem, and nicergoline) compounds. Through a systematic study, they showed that such an approach could be greener for pharmaceutical applications without any major impact in terms of elution order, chromatographic separation, efficiency, and peak symmetry. Moreover, the study of thermodynamic parameters showed that partition of analytes between the mobile and stationary phases was similar for both solvent systems. However, the most important difference is a lower mass transfer of analytes using PC-based mobile phases, so that optimal flow rates were found to be lower compared to acetonitrile-based mobile phases, increasing analysis time. Also, the limited miscibility of PC and pressure drop should be taken into account.

Acetonitrile replacement by PC/EtOH mixtures has also been applied to bioanalytical applications by Cheregi et al. [60]. A LC/MS–MS assay of enalapril and enalaprilat in human plasma was developed, and similar chromatographic performance was achieved using this green approach compared to the reference one. In addition, Dogan et al. [64] developed and validated green bioanalytical HPLC methods for voriconazole and tadalafil analysis using a mobile phase composed of a mixture of PC and EtOH as organic modifiers. 

Finally, PC appears to have the same advantages as EtOH, with the additional disadvantage coming from its limited miscibility with water. Indeed, the use of PC implies a ternary mobile phase leading to more difficult developments. It may explain the lower number of applications found with PC compared to EtOH.

### 2.3. Others Solvents

#### 2.3.1. Acetone

Acetone is another greener substitute to ACN in RP-HPLC applications due to its low toxicity and its biodegradability properties. Acetone shares similar physicochemical characteristics to ACN in terms of solubility and miscibility properties with other solvents, including water. Indeed, acetone is in the same group as ACN in Snyder et al.’s [2] classification. In addition, they present similar viscosities (0.37 mPa·s for ACN and 0.33 mPa·s for acetone at 20 °C) [26]. The main drawback of acetone compared to ACN is its high UV cut-off, which reaches 340 nm, limiting its use with UV detection [9]. Moreover, acetone is highly volatile, and therefore difficult to pump. However, the fast popularization of MS-based detectors in LC and the new generation of aerosol-based LC detectors offer new opportunities for the usage of acetone in RP-HPLC. 

The possibility of effectively substituting ACN with acetone was investigated by Funari et al. [65]. In this study, a RP-HPLC method with double detection UV and corona-charged aerosol detector (CAD) was developed to fingerprint a complex plant extract. Based on the design of experiments, the methods using ACN or acetone were successfully optimized and compared. The same performance of separation and detection was noticed with both solvents using a CAD detector. The number of detected peaks and peak capacity were proved to be statistically similar. However, the superiority of acetone was evidenced when parameters relating to method greenness were taken into account. Moreover, there are some reports of satisfactory replacement of ACN with acetone in RP-HPLC employing mass spectrometer detectors in the analysis of peptides [66,67]. However, as UV remains the most widespread detector in pharmaceutical analysis, acetone is clearly not the favorite green solvent to replace ACN in RP-HPLC.

#### 2.3.2. Ethyl Lactate

Ethyl lactate is industrially produced as a racemic mixture from the reaction of EtOH with lactic acid, wherein water is the sole by-product. Thus, ethyl lactate has been well recognized as an environmentally benign solvent that possesses unique advantages, such as low production cost, non-toxicity (used as an additive in the food industry), high biodegradability, and excellent miscibility with water and others organic compounds [68].

Ethyl lactate has been studied as an environmentally friendly organic modifier to be used in HPLC mobile phases [27,69]. For example, Judge et al. [69] showed that a mobile phase composed of 87% water, 10% ethyl lactate, and 3% acetic acid allowed a baseline separation of three standard pharmaceutical analytes (acetaminophen, caffeine, and acetylsalicylic acid) on a standard C18 column at a temperature of 60 °C under three minutes. However, the use of ethyl lactate has some major drawbacks limiting its use in RP-HPLC, such as its chemical stability when using acidic or alkaline conditions in the mobile phases, its cut-off wavelength significantly much higher than that of ACN, its lack of availability on the market in a chromatographic grade, and its viscosity even if it produces pressure drops compatible with conventional LC instruments [27].

#### 2.3.3. Ethyl Acetate

Ethyl acetate is another green solvent which can be used in mobile phases to replace ACN and MeOH [26]. However, this solvent has some drawbacks such as its high UV cut-off wavelength (256 nm), its low miscibility with water, and its chemical instability, particularly with acids and bases [2,26]. Despite these limits, an environmentally benign RP-HPLC method with a mobile phase composed of 100% ethyl acetate was developed and validated by Haq et al. [70] for the analysis of indomethacin in bulk drugs, nanoemulsions, and various pharmaceutical formulations. Also, the same authors developed and validated a RP-HPLC method using a mobile phase composed of ethyl acetate/EtOH (50:50, *v*/*v*) for a rapid analysis of olmesartan medoxomil in bulk drugs, self-microemulsifying drug delivery systems, and marketed tablets [71].

## 3. Aqueous Mobile Phases

Using 100% aqueous mobile phases is a good way to make conventional RP-HPLC greener, as water is one of the most benign solvents. Indeed, water is readily available, inexpensive, non-toxic, non-flammable, and environmentally friendly with no disposal concerns. In addition, water does not absorb in UV down to 190 nm, so that it is possible to detect even weak chromophores [72]. Also, using water as an eluent allows the combination of LC with non-conventional detectors, (e.g., flame ionization detector (FID)) as water has no significant FID response. This is highly desirable for UV-transparent compound analysis. Moreover, the compatibility of heated water with MS detectors has been shown to increase the ionization efficiency and signal-to-noise ratio (S/N) for some compounds [17]. 

Some applications report the use of totally aqueous mobile phases in RP-HPLC at relatively low temperatures for the analysis of pharmaceuticals [73,74]. Nevertheless, such applications are limited to the separation of highly polar analytes since, in RP-HPLC, water is the weakest solvent due to its high polarity (dielectric constant, ε = 80 at 20 °C) [72]. Indeed, the elution strength of water at ambient temperature is usually not sufficient to elute relatively non-polar compounds in RP-HPLC. Another problem coming from the use of totally aqueous mobile phases is the phenomenon of phase collapse or phase dewetting, which occurs with alkyl bonded phases (such as C8 or C18) in RP-HPLC [75]. In fact, phase dewetting leads to chromatographic problems such as retention loss, peak tailing, non-reproducible retention times, and gradient regeneration delays. Nevertheless, this issue of phase collapse can be avoided by using columns especially designed to be compatible with highly aqueous environments (i.e., including a polar end-capping or polar embedded groups) [75]. For example, a RP-HPLC-DAD method was developed and validated using only aqueous solvents, by Langlois et al. [73] for paracetamol quantitation in cell culture fluid from an in vitro Blood Brain Barrier model using a C18 column with embedded polar groups (XTerra RP18 column) at 35 °C. The mobile phase was a 20 mM formate buffer in water at pH 4. Also, Šatínský et al. [74] have developed and validated a simple, rapid, and environmentally friendly method for the separation of four compounds (4-aminophenol, caffeine, paracetamol, and propyphenazone) on a polyethylene glycol (PEG) stationary phase at 30 °C, with a low-toxicity mobile phase consisting of water and 0.04% (*v*/*v*) trimethylamine, whose pH was adjusted to 4.5 by means of glacial acetic acid. Then, the proposed green method was successfully applied to the analysis of active substances and one degradation product (4-aminophenol) in commercial preparations.

Due to its low elution strength at room temperature, water cannot elute many organic analytes, thus, high temperatures are usually needed to increase its elution strength. Indeed, at elevated temperatures, the dielectric constant of water decreases, increasing its elution strength [34]. Chromatographic separation performed with water at high temperatures is known as superheated water chromatography (SHWC), or subcritical water chromatography (SWC) when the temperatures used are lower than the critical temperature of water (374 °C) [76]. At high temperature, pure water tends to have a similar polarity as eluents typically used in RP-HPLC [72]. For example, at 150 °C, the dielectric constant of water is reduced, and its elution strength becomes comparable to that of a mixture of MeOH/water 50:50 (*v*/*v*) at ambient temperature [34].

Some comprehensive reviews about SHWC have been published [72,76,77]. As does HTLC, SHWC requires special instrumentation, such as a mobile phase preheater, a column heater, and a post-column effluent cooling system [34]. Also, the most used silica-based stationary phases in RP–LC are not stable at temperatures above 50 °C, since the reactive properties of water at high temperatures accelerates the dissolution of silica [17,33]. More temperature-resistant packing materials are usually required for SHWC, such as polymeric phases (polystyrene-divinylbenzene (PS-DVB)) or zirconia-based materials (e.g., zirconia particles with polybutadiene (PBD), polystyrene, or a carbon coating) [76]. Moreover, SHWC has other limitations, such as potential on-column degradation of thermally labile analytes and insolubility of hydrophobic analytes in pure water [3,35].

Several applications of SHWC in pharmaceutical analysis have been reported in the literature [78,79,80,81,82,83]. As examples, Fields et al. [82] showed that the replacement of ACN/water mixtures with superheated water mobile phases in RP-HPLC can be successfully applied to the analysis of testosterone and several related compounds on a porous zirconia, PBD-coated column at temperatures up to 200 °C. Six anticancer drugs were also well separated on a PS-DVB column at 160 °C, with a buffered superheated water as the mobile phase [80]. In another study, a SHWC method was established for the analysis of pharmaceutical compounds in cold drugs, in substitution of traditional RP-HPLC [78]. A mixture of pure water and 100 mM phosphoric acid was used as the mobile phase on an Alltech Adsorbosil C18 column. To optimize the separation of pharmaceuticals, a gradient elution based on an increase in temperature was programmed. Also, Huang et al. [83] compared the chromatographic analysis of eleven thiazide and related sulfonamide diuretics on an XBridge C18 column using SHWC up to 200 °C, and conventional liquid chromatography at ambient temperature. Most of the sulfonamide diuretics were thermally stable, but many of the thiazides were degraded. The results confirmed that the thermal stability of analytes must be considered before using SHWC.

## 4. Micellar Liquid Chromatography

Micellar liquid chromatography (MLC) is a reversed-phase liquid chromatographic mode in which the stationary phase is nonpolar and the mobile phase is an aqueous solution of a surfactant at a concentration above the critical micellar concentration (CMC) [84]. In comparison with aqueous-organic mobile phases, the addition of a surfactant to the mobile phase in RP-HPLC modifies the chromatographic behavior, since it introduces a pseudo-stationary phase into which analytes can partition. In MLC, analyte retention depends on three phases: stationary phase, bulk solvent, and micellar pseudophase. Compounds are therefore separated based on their differential partitioning between these three phases. Most of the time, surfactants are added to the mobile phase to form micelles, but surfactant-coated stationary phases can also be used. For water-insoluble species, partitioning mainly occurs via direct transfer of analytes between the micellar pseudophase and the stationary phase [85]. Different papers with comprehensive overviews of MLC have been published [84,85,86,87].

Micellar liquid chromatography has been investigated as an interesting approach for GAC, as it eliminates or reduces the use of organic solvents. Indeed, mobile phases contain aqueous solutions of a surfactant and a small percentage of organic modifier (mostly below 15%, *v*/*v*). Moreover, these micellar mobile phases are non-flammable, have a low toxicity, and do not produce hazardous waste because of the biodegradable character of the surfactants used [88]. For example, sodium dodecyl sulfate (SDS), the most commonly used surfactant in MLC, is a fatty alcohol sulfate that is aerobically degraded [89,90,91]. However, it is often necessary to add organic solvents to the aqueous solutions of micelles in order to improve MLC separations [86]. The most commonly used organic modifiers in MLC are propanol, butanol, and pentanol, which are less toxic than MeOH or ACN [4]. Another important advantage of MLC concerns sample treatment. In fact, the great solubilizing ability of micelles allows the direct injection of drugs in complex matrices (e.g., biological fluids and dosage forms) without the need for sample pretreatment other than filtration [84]. Moreover, MLC is compatible with existing RP-HPLC instruments. Thus, it does not require any modification of existing RP-HPLC instrumentation.

Table 2 lists the different, recent applications of MLC for analysis of drugs in both pharmaceutical and biological matrices [92,93,94,95,96,97,98,99,100,101,102,103,104,105,106,107,108,109,110,111,112,113,114,115,116,117,118]. Although most of these methods used SDS as a surfactant, some of them also used Brij-35 [92,93], cetyltrimethylammonium bromide (CTAB) [94], or Tween-20 [95]. In the majority of applications, hybrid micellar liquid chromatography was reported using mobile phases composed of an aqueous surfactant solution and a small volume of organic modifiers, such as n-propanol, n-butanol, and n-pentanol. As an example, El-Shaheny [96] developed and validated a stability-indicating MLC method for the determination of three oxicams (piroxicam, tenoxicam, and lornoxicam) using a C8 column, and a mobile phase composed of 0.15 M SDS with 10% propanol. This method was applied to quality control of pharmaceutical preparations, including gel and suppositories, without pretreatment steps other than dilution and filtration. Another MLC method was established to quantify three selective serotonin reuptake inhibitors (citalopram, paroxetine, and fluoxetine) using a mobile phase buffered at pH 7, containing SDS and 6% (*v*/*v*) butanol [97]. The suitability of the method was then successfully checked through the analysis of tablets and biological samples (i.e., plasma and urine samples), without extraction steps from patients treated with selective serotonin reuptake inhibitors.

There are also some interesting examples of green MLC analysis of pharmaceuticals using totally aqueous mobile phases [92,93,98,99]. A MLC method using a 0.05 M SDS aqueous solution at pH 7 as a mobile phase was developed and validated by Peris-Vicente et al. [99] to quantify abacavir, lamivudine, and raltegravir in plasma. Also, Fernández-Navarro et al. [92] successfully analyzed seven tricyclic antidepressants in pharmaceutical formulations using 0.02 M of Brij-35 in the mobile phase. The preparation of the samples required only solubilization and filtration steps previous to injection.

To conclude, MLC is an interesting alternative to develop green chromatographic methods for pharmaceutical analysis. Moreover, MLC enables the simplification of sample preparation for complex biological sample analysis. Also, it can be hyphenated with UV, fluorescence, and even electrochemical detections. However, such methods involve a high number of parameters, among them the type and the concentration of surfactants, and the nature and the percentage of organic solvents, leading to quite difficult chromatographic developments. Indeed, due to the complexity of the mobile phase, retention mechanisms are often difficult to predict or to interpret.

## 5. Ionic Liquids as Green Mobile Phase Additives

Ionic liquids (ILs), which are also called room-temperature ionic liquids (RTILs), are liquid at ambient temperature, and are composed entirely of ions, most often organic cations, but also inorganic or organic anions [119]. These low-melting-point salts (below 100 °C) were first intensively used as solvents for organic synthesis but have more recently shown great potential as green solvents for analytical chemistry in academic and industrial fields. Indeed, they possess beneficial properties, such as high miscibility with water and organic solvents, capacity to solubilize organic and inorganic compounds, high thermal stability, negligible vapor pressure (low volatility), and non-flammability [120,121]. However, their environmentally-friendly nature was recently questioned as aquatic toxicity is a matter of concern with respect to the environmental safety of ILs [16].

A review of applications of ionic liquids in the whole field of analytical chemistry has been published [120]. Other reviews focus on applications in separation sciences [121,122], and some specifically on liquid chromatography [119,123,124]. In chromatographic separations, ionic liquids are most common used as mobile phase additives, and sometimes as surface-bonded stationary phases for ion-exchange (IEX) or mixed mode IEX/RP-HPLC [119,122]. ILs are usually used in small quantity in RP-HPLC mobile phases as a silanol-blocking agent to improve the chromatographic analysis of basic compounds. In fact, ILs can reduce or suppress interactions between positively charged compounds and anionic free silanols of the stationary phase at low pH mobile phases, which are responsible for peak tailing and peak broadening. ILs can also be added in higher proportions to totally aqueous mobile phases instead of polluting organic modifiers.

Some applications of ILs as mobile phase additives in RP-HPLC have been reported for the analysis of pharmaceutical compounds [125,126,127,128,129,130]. Different pharmaceutical compounds were analyzed such as β-blockers [125], antidepressants [126], β-lactam antibiotics [127], thiamine (vitamin B1) [128], urazamide [129], and ephedrines [130]. Among these, Ruiz-Angel et al. [125] carried out a comparative study of the effect of 1-butyl-3-methylimidazolium tetrafluoroborate ([BMIM][BF4]) and triethylamine (TEA) added to the mobile phase on the analysis of seven β-blockers in RP-HPLC. They showed that, in terms of efficiency and asymmetry factor, BMIM BF4 was a significantly better additive compared to TEA. Even if this study highlights the benefits of using ILs, the greenness of the method can be questioned, as 30% ACN was used in the mobile phase. Also, Seo et al. [129] developed and successfully validated a green method for the determination of urazamide in pharmaceutical preparation using an aqueous mobile phase modified with 1-ethyl-3-methyl-imidazolium tetrafluoroborate.

## 6. Conclusions

Greening liquid chromatographic methods have become of great interest to the field of pharmaceutical analysis to protect both the operators’ health and the environment. Indeed, thousands of chromatographic equipment are routinely used for quality control of pharmaceuticals around the world, representing high amounts of organic solvents consumed and wastes generated.

In this review, we have shown that a good strategy to make RP-HPLC methods greener for analysis of pharmaceuticals is the replacement of the toxic solvents commonly used, mainly acetonitrile and methanol, with more benign alternative solvents. Many strategies have been described. Among them, the use of ethanol as a green organic solvent in the mobile phase is probably the most promising one, as it is economical and follows a classical development in RP-HPLC. Totally aqueous mobile phases at high temperatures or modified with surfactants or ionic liquids are also interesting approaches.

The different applications presented herein highlight that green RP-HPLC methods using alternative solvents can be successfully performed without any major compromise in terms of chromatographic performance. Moreover, these approaches are rather economical, as they do not require expensive equipment and reduce waste disposal costs. Consequently, they are expected to develop in the coming years.

## Figures and Tables

**Table 1:** Examples of pharmaceutical analysis in RP-HPLC using ethanol-based mobile phases.

Compounds	Matrix	Column	Mobile Phase	Flow Rate (mL/min)	Temperature (°C)	Detection	Ref
Prednisolone	Tablets	Phenomenex C18 (150 × 4.6 mm, 5 μm)	EtOH/water (30:70, *v*/*v*)	0.8	50	UV (254 nm)	[38]
Diltiazem	Topical formulations	C18 column (250 × 4.6 mm, 5 μm)	EtOH/H_3_PO_4_ (pH 2.5) (35:65, *v*/*v*)	2	50	UV (240 nm)	[39]
Permethrin isomers	Pharmaceutical formulations (cream)	C18 column (150 × 4.6 mm, 5 μm)	EtOH/H_3_PO_4_ (pH 3.0) (67:33, *v*/*v*)	1	30	UV (215 nm)	[40]
Ampicilline sodium	Powder for injection solution	Zorbax C18 (150 × 4.6 mm, 5 μm)	EtOH/water (40:60, *v*/*v*)	0.5	25	UV (210 nm)	[41]
Statins: pravastatin; fluvastatin; atorvastatin	Pharmaceutical hydro-alcoholic solutions	ODS-AQ YMC C18 (50 × 4.6 mm, 3 µm)	EtOH/25 mM formic acid (pH 2.5) (50:50, *v*/*v*)	1.0	40	UV (238 nm)	[42]
Daptomicin	Lyophilized powder	Zorbax C18 (150 × 4.6 mm, 5 μm)	EtOH/water (55:45, *v*/*v*) pH 4.5 with glacial acetic acid	0.6	25	UV (221 nm)	[43]
Dextromethorphan and its impurities	Pharmaceutical products	Acquity BEH C18 (50 × 2.1 mm, 1.7 μm)	Gradient elution of EtOH/10 mM ammonium formate buffer (pH 4.7)	0.23	38	UV (280 nm)	[44]
Mebeverine hydrochloride/sulpiride	Coformulated pharmaceuticals	Zorbax SBC18 (75 × 4.6 mm, 3.5 µm)	EtOH/water (94.5:5.5, *v*/*v*)	0.8	room temperature	UV (220 nm)	[45]
Chlorphenoxamine hydrochloride/caffeine/chlorotheophylline	Coformulated pharmaceuticals	Polaris SI (50 × 4.6 mm, 3 µm)	EtOH (100%)	0.4	room temperature	UV (220.4 nm, 270.4 nm, and 276.4 nm)	[45]
Clidinium bromide/chlordiazepoxide hydrochloride; phenobarbitone/pipenzolate bromide	Coformulated pharmaceuticals	Zorbax SBC18 (75 × 4.6 mm, 3.5 µm)	EtOH/water (50:50, *v*/*v*)	0.5	room temperature	UV (210 nm and 220 nm)	[45]
Telmisartan; hydrochlorothiazide; amlodipine besylate	Tablets	Intersil ODS-3 C18 (250 × 4.6 mm, 5 μm)	EtOH/20 mM phosphate buffer (pH 7) (70:30, *v*/*v*)	0.7	25	UV (240 nm)	[46]
Phenylephrine; Paracetamol; Guaifenesin	Tablets	Onyx Monolithic C18 (100 × 4.6 mm)	Gradient elution of EtOH/phosphate buffer (pH 7)	2	Room temperature	UV (220 nm)	[47]
Cefepime hydrochloride	Lyophilized powder for solution for injection	Luna C18 (250 × 4.6 mm, 5 μm)	EtOH/water (55:45, *v*/*v*)	0.5		UV (258 nm)	[48]
Lansoprazole enantiomers		Stainless-steel Chiralpak IC-3 (100 × 4.6 mm)	EtOH/water (50:50, *v*/*v*)	1.0	40	UV (210 nm and 280 nm)	[49]
Dapsone	Pharmaceutical preparations	C18	EtOH/formic acid (pH 3) (10:90, *v*/*v*)			UV detection	[50]
Caffeic acid	Pharmaceutical products	RP18 XDB Waters (250 × 4.6 mm, 5 μm)	EtOH/acetic acid (pH 2.5) (40:60, *v*/*v*)	0.7	25	UV (325 nm)	[51]
Ertapenem sodium	Powder for injection solution	Zorbax Bonus-RP (150 × 4.6 mm, 5 μm)	EtOH/0.1% formic acid (20:80, *v*/*v*)	1.0		UV (297 nm)	[52]
Rifaximin	Tablets	Eclipse Plus C18 (150 × 4.6 mm)	EtOH/0.1% glacial acetic acid (52:48, *v*/*v*)			UV (290 nm)	[53]
16 active pharmaceutical ingredients		Xbridge BEH Shield RP18 (50 × 4.6 mm, 2.5 µm)	Gradient elution of EtOH/20 mM acetate buffer (pH 4.85)	0.8	33.7	UV (210 nm)	[54]
Quetiapine	Rat plasma	Acquity BEH C18 (50 × 2.1 mm, 1.7 μm)	EtOH/water/formic acid (80:20:0.1, *v*/*v*/*v*)	0.3	40	MS/MS detection	[55]
Capecitabine	Human plasma	C18 RP column	EtOH/formic acid (pH 3) (55:45, *v*/*v*)	1.0	50	UV (310 nm)	[56]

**Table 2:** Some recent applications of MLC in pharmaceutical analysis.

Compounds	Matrix	Column	Mobile Phase	Temperature (°C)	Detection	Ref
Seven tricyclic antidepressants	Pharmaceutical formulations	Zorbax C18 (150 × 4.6 mm)	0.02 M Brij-35 with citric buffer, pH 3.0 1 mL/min	25	UV (254 nm)	[92]
Six β-blockers		Zorbax Eclipse C18 (150 × 4.6 mm, 5 μm)	0.02 M Brij-35 and 0.15 M SDS with 0.01 M NaH_2_PO_4_ and HCl, pH 3.0 1 mL/min	25	UV (225 nm)	[93]
Seven tricyclic antidepressants		Zorbax Eclipse C18 (150 × 4.6 mm, 5 μm)	0.02 M Brij-35 with 0.01 M NaH_2_PO_4_ and HCl, pH 3.0 1 mL/min	25	UV (254 nm)	[93]
Free ampicillin	Standard solutions of human serum albumin	RP-8 endcapped column (125 × 4.0 mm, 5 μm)	0.06 M CTAB + 20% ACN (*v*/*v*), pH 7.4 1 mL/min	20	UV (254 nm)	[94]
Nelfinavir mesylate	Tablets	Licrosphere c18	0.5 M Tween-20 + 2% *n*-butanol (*v*/*v*) with H_3_PO_4_, pH 4.2 1.5 mL/min	25	UV (249 nm)	[95]
Piroxicam, tenoxicam, and lornoxicam	Gel and suppositories	C8 column (150 × 4.6 mm, 5 µm)	0.15 M SDS + 10% *n*-propanol (*v*/*v*) with 0.3% TEA and 0.02 M H_3_PO_4_, pH 3.0		Time-programmed UV detection	[96]
Citalopram, paroxetine, and fluoxetine	Plasma and urine	Kromasil C18 (150 × 4.6 mm, 5 μm)	0.075 M SDS + 6% *n*-butanol (*v*/*v*) with 0.01 M NaH_2_PO_4_, pH 7.0 1 mL/min	25	Fluorescence detection program	[97]
Sildenafil citrate	Oral suspensions and tablets	ACE 5 C18–AR column (50 × 4.6 mm, 5 µm)	0.0082 M SDS with acetate buffer, pH 4.0 0.5 mL/min	room temperature	UV (298 nm)	[98]
Abacavir, lamivudine, and raltegravir	Human plasma	Kromasil C18 (150 × 4.6 mm, 5 μm)	0.05 M SDS, pH 7.0 1 mL/min	room temperature	UV (260 nm)	[99]
Nicotine	Pharmaceutical formulations and biological fluid	Kromasil C18 (250 × 4.6 mm, 5 µm)	0.15 M SDS + 6% *n*-pentanol (*v*/*v*) with 0.01 M NaH_2_PO_4_ and 0.001 M KCl, pH 6.0		Electrochemical detector (0.8 V) UV (259 nm)	[100]
Tamoxifen	Plasma of breast cancer patients	Kromasil 5 C18 (150 × 4.6 mm, 5 µm)	0.15 M SDS + 7% *n*-butanol (*v*/*v*), pH 3.0 1.5 mL/min	40	Fluorescence (260 nm/380 nm)	[101]
Penicillin antibiotics (amoxicillin, ampicillin, cloxacillin, and dicloxacillin)	Pharmaceutical formulations and physiological fluids (urine)	Zorbax C18 (150 × 4.6 mm, 5 µm)	0.11 M SDS + 6% *n*-propanol (*v*/*v*) with 0.01 M NaH_2_PO_4_, pH 3.0 1 mL/min	25	UV (210 nm)	[102]
Tamoxifen and endoxifen	Plasma samples from breast cancer patients	Kromasil C18 (150 × 4.6 mm, 5µm)	0.15 M SDS + 7% *n*-butanol (*v*/*v*), pH 3.0 1.5 mL/min	40	Fluorescence (260 nm/380 nm)	[103]
Morphine, codeine, papaverine, and noscapine	Pharmaceutical solution for injection	Kromasil C18 (150 × 4.6 mm, 5 μm)	0.10 M SDS + 5% *n*-butanol (*v*/*v*) with H_3_PO_4_, pH 2.5 1 mL/min	40	UV (280 nm)	[104]
Derivatives of zidovudine	Simulated gastric and intestinal fluids	Phenomenex Synergi Fusion-RP 80 (250 × 4.6 mm, 4 μm)	0.05 M SDS + 1% *n*-butanol (*v*/*v*) with 0.01 M NaH_2_PO_4_, pH 3.0 1 mL/min	30	UV (267 nm)	[105]
Lamivudine and its carbonate derivatives	Simulated gastric and intestinal fluids	Kromasil C18 (250 × 4.6 mm, 5 μm)	0.15 M SDS + 4% *n*-butanol (*v*/*v*) with 0.01 M KH_2_PO_4_-Na_2_HPO_4_, pH 7.0 1 mL/min	30	UV (272 nm)	[106]
Flavoxate hcl	Tablets	BDS Hypersil phenyl (250 × 4.6 mm, 5 µm)	0.15 M SDS + 15% *n*-propanol (*v*/*v*) with 0.3% TEA and 0.02 M H_3_PO_4_, pH 2.5 1 mL/min		UV (325 nm)	[107]
Diltiazem hydrochloride, metoprolol tartrate, and isosorbide mononitrate	Human serum	Pinnacle II Cyano column (150 × 4.6 mm, 5 µm)	0.0415 M SDS + 10% *n*-propanol (*v*/*v*) with 0.02 M NaH_2_PO_4_, pH 7.0 0.8 mL/min	40	UV (225 nm)	[108]
Tizoxanide	Human urine and plasma	Chromolith^®^ C18 (100 × 4.6 mm)	0.1 M SDS + 8% *n*-propanol (*v*/*v*) with 0.3% TEA and 0.02 M H_3_PO_4_, pH 4.0 1.0 mL/min	25	UV (240 nm)	[109]
β-blockers (acebutolol, atenolol, carteolol, labetolol, metoprolol, and propranolol , celiprolol, and oxprenolol)	Urine samples	Zorbax Eclipse XDB-C8 and Zorbax Eclipse XDB-C18 (150 × 4.6 mm, 5 µm)	Gradient mode: 0.10 M SDS + *n*-propanol (from 0 to 30% (*v*/*v*) in 15 min) 1 mL/min	25	UV (225 nm)	[110]
Felodipine	Tablets and human plasma	Shim-pack CLC-C18 (250 × 4.6 mm, 5 μm)	0.085 M SDS + 6.5% *n*-pentanol (*v*/*v*) with 0.025 M phosphate buffer, pH 7.0 1.5 mL/min	30	Fluorescence (240 nm/440 nm)	[111]
Darunavir, ritonavir, emtricitabine, and tenofovir	Human plasma	Kromasil C18 column (150 × 4.6 mm, 5 μm)	0.06 M SDS + 2.5% *n*-pentanol (*v*/*v*), pH 7.0 1 mL/min	room temperature	UV (214 nm)	[112]
Ascorbic acid, pseudoephedrine hydrochloride, and ibuprofen	Tablets	ODS C18 stainless steel (150 × 4.6 mm)	0.03 M SDS + 8% 1-propanol (*v*/*v*) with 0.3% TEA and 0.02 M H_3_PO_4_, pH 3.0 1 mL/min		UV (260 nm)	[113]
Ofloxacin and flavoxate	Pharmaceutical formulations	BDS Hypersil phenyl (250 × 4.6 mm, 5 μm)	0.15 M SDS + 15% n-propanol (*v*/*v*) with 0.3% TEA and 0.02 M H_3_PO_4_, pH 2.5 1 mL/min		UV (325 nm) and fluorescence (290 nm/485 nm)	[114]
Tamoxifen and its main metabolites	Plasma samples from breast cancer patients	C18 column	0.08 M SDS + 4.5% *n*-butanol (*v*/*v*), pH 3.0 1.5 mL/min	40	Fluorescence (260 nm/380 nm)	[115]
Enalapril maleate, lisinopril dihydrate, benazepril hydrochloride, and hydrochloro-thiazide	Tablets	C18 silica column	0.012 M SDS + 10% *n*-propanol (*v*/*v*) with 0.3%TEA and 0.02 M H_3_PO_4_, pH 3.6		UV (210 nm)	[116]
Esomeprazole, leflunomide, and ibuprofen	Human plasma and tablets	Shim-pack VP-ODS stainless steel column (150 × 4.6 mm)	0.1 M SDS + 10% *n*-propanol (*v*/*v*) with 0.3% TEA and 0.02 M H_3_PO_4_, pH 3.5 1 mL/min		UV (285 nm)	[117]
Axitinib, lapatinib, and afatinib	Plasma	Kromasil C18 column (150 × 4.6 mm, 5 μm)	0.07 M SDS + 6.0% *n*-pentanol (*v*/*v*) with 0.01 M phosphate salt, pH 7.0		UV (260 nm)	[118]

ACN, acetonitrile; CTAB, cetyltrimethylammonium bromide; SDS, sodium dodecyl sulfate; TEA, triethylamine.
